# The role of magnetic resonance imaging in active surveillance of prostate cancer

**DOI:** 10.1590/0100-3984.2020.0069

**Published:** 2021

**Authors:** Olayemi Atinuke Alagbe, Antonio Carlos Westphalen, Valdair Francisco Muglia

**Affiliations:** 1 Faculdade de Medicina de Ribeirão Preto - Universidade de São Paulo (FMRP-USP), Ribeirão Preto, SP, Brazil.; 2 School of Medicine, University of California San Francisco (UCSF), San Francisco, CA, USA.

**Keywords:** Prostatic neoplasms/diagnostic imaging, Watchful waiting/methods, Magnetic resonance imaging/methods, Neoplasms/diagnostic imaging, Neoplasias da próstata/diagnóstico por imagem, Conduta expectante/métodos, Ressonância magnética/métodos, Neoplasias/diagnóstico por imagem

## Abstract

Active surveillance (AS) is an important strategy to avoid overtreatment of prostate cancer (PCa) and has become the standard of care for low-risk patients. The role of magnetic resonance imaging (MRI) in AS has expanded due to its ability to risk stratify patients with suspected or known PCa, and MRI has become an integral part of the AS protocols at various institutions. A negative pre-biopsy MRI result is associated with a very high negative predictive value for a Gleason score ≥ 3+4. A positive MRI result in men who are otherwise eligible for AS has been shown to be associated with the presence of high-grade PCa and therefore with ineligibility. In addition, MRI can be used to guide and determine the timing of per-protocol biopsy during AS. However, there are several MRI-related issues that remain unresolved, including the lack of a consensus and guidelines; concerns about gadolinium deposition in various tissues; and increased demand for higher efficiency and productivity. Similarly, the need for the combined use of targeted and systematic sampling is still a matter of debate when lesions are visible on MRI. Here, we review the current AS guidelines, as well as the accepted roles of MRI in patient selection and monitoring, the potential uses of MRI that are still in question, and the limitations of the method.

## Abstract (pt)

A vigilância ativa (VA) é uma estratégia importante para evitar o tratamento excessivo do câncer de próstata (CaP) e tornou-se o padrão de atendimento a pacientes de baixo risco. O papel da ressonância magnética (RM) na VA tem se expandido, devido à sua capacidade de estratificar o risco pacientes com CaP suspeito ou diagnosticado, tornando-se parte integrante dos protocolos de VA em várias instituições. Uma RM pré-biópsia negativa está associada a um valor preditivo negativo muito alto para o diagnóstico de Gleason ≥ 3+4. Um exame positivo em homens que são elegíveis para VA tem se mostrado associado à presença de CaP de alto grau e inelegibilidade para VA. A RM também pode ser usada para orientar e determinar o tempo ideal de uma biópsia, ou por protocolo, durante a VA. Há, no entanto, várias questões relacionadas à RM que permanecem não resolvidas. Estas incluem a falta de consenso ou diretrizes, preocupações com o depósito de gadolínio em vários tecidos e aumento da pressão por maior eficiência e produção. Da mesma forma, a necessidade de biópsia sistemática combinada à dirigida continua a ser uma questão controversa, quando as lesões são visíveis na RM. Revisaremos as atuais diretrizes de VA, os papéis consensualmente aceitos da RM na seleção e monitoramento dos pacientes, potenciais usos, ainda discutíveis, e as limitações do método.

## INTRODUCTION

Prostate cancer (PCa) is the most common type of cancer, with a worldwide prevalence of 25%, and is the second leading cause of cancer death in men^(1)^. However, in most cases, PCa has an indolent course and does not result in clinically significant disease. Although some carcinomas progress rapidly to a life-threatening condition, a small fraction of clinically significant cancers remain confined to the prostate for many years^(2)^.

Active surveillance (AS) is a strategy that is increasingly being accepted as a viable management option aimed at postponing curative therapy for patients with low-risk disease until evidence of cancer progression is detected^(3,4)^. Although the AS patient eligibility criteria and monitoring protocols vary widely^(5-8)^, as detailed in Table 1, they often include total serum prostate specific antigen (PSA), clinical T stage, Gleason score, total number of positive biopsy cores, percentage of the length of biopsy cores affected by cancer, the time intervals for patient monitoring, and life expectancy.

**Table 1:** Main AS protocols.

Protocol	Gleason score	PSA (ng/ml)	Clinical stage	Positive scores (n or %)	PSA density (ng/mL^2^)	Follow-up interval
NICE**^181^**	*<* 6	< 10	T1-T2a	NA	NA	PSA every 3-4 months in the first year and every 3-6 months (second year). Repeat TRUS-guided biopsy at 12 months
PRIAS**^161^**	*< 6*	< 10	T1c-T2	< 2	< 0.2	PSA every 3 months for the first 2 years, then every 6 months. Repeat TRUS-guided biopsy at 12 months, 4 years, and 7 years. If the PSA doubles, repeat annually for up to 10 years
UCSF**^171^**	*< 6*	< 10	T1c-T2	< one-third of the total needle samples and < 50% of any single needle sample	NA	PSA every 3 months. Repeat TRUS-guided biopsy every 1-2 years
CCO**^1421^**	*< 6*	< 10	< T2a	NA	NA	PSA every 3-6 months. TRUS-guided biopsy after 6 or 12 months (in year 1). Thereafter, serial biopsy every 3-5 years

NICE, National Institute for Health and Care Excellence; PRIAS, Prostate Cancer Research International: Active Surveillance (PRIAS) study; UCSF, University of Cali­fórnia San Francisco; CCO, Cancer Care Ontario; NA, not applicable.

To date, studies examining the role of prostate magnetic resonance imaging (MRI) have focused on diagnosis, staging, and detection of local recurrence after treatment^(9-11)^. Its use in the selection and monitoring of patients under AS has not been fully defined. One recent review of 30 large AS cohort studies showed that MRI was used as an adjuvant to other AS selection criteria in only two of those studies^(9)^. Nonetheless, MRI is becoming an increasingly more important tool for assessing patients who are being considered for enrollment in AS.

## ROLE OF MRI

### Patient selection and enrollment

Low-risk and very low-risk PCa patients are generally considered to be eligible for AS. Among the criteria used to determine the risk of a patient with PCa, the concept of clinically insignificant disease is one of the most important. Although several definitions of clinically insignificant disease are found in the literature, many are aligned with the definition proposed by Epstein et al. in 1994^(12)^: organ-confined disease; Gleason score ≤ 6; no Gleason pattern 4 or 5; and tumor volume less than 0.5 cm^3^ (approximate long-axis diameter < 1.0 cm).

The great dilemma is that PCa is a heterogeneous disease and the adoption of any conservative management approach requires a high probability that tumors with aggressive behavior have been excluded. This is often done by combining that approach with other tests, such as genetic counseling and MRI. It has been demonstrated that MRI has incremental predictive value when used in combination with clinical AS eligibility criteria^(13)^, as well as that it is effective in predicting reclassification of patients with low-risk PCa before enrollment in AS^(14)^. It is specifically suitable to identify patients with high-grade, high-volume disease who would benefit from subsequent treatment and to reduce unnecessary evaluation and treatment of patients with low-grade, low-volume disease^(15)^.

Current guidelines, including those of the European Association of Urology^(16)^ and Prostate Imaging Reporting and Data System (PI-RADS), recommend a multiparametric MRI (mpMRI) protocol-combining high-resolution T2-weighted images, diffusion-weighted imaging (DWI), and dynamic contrast-enhanced imaging-for men under AS, irrespective of suspected disease progression^(17)^. When performed at the time of AS enrollment, the risk of clinically significant disease in patients with a negative mpMRI result may be sufficiently low to allow such patients to be considered eligible for AS^(18)^. The study conducted by Villers et al.^(19)^ indicated that a negative mpMRI virtually excludes clinically significant cancer.

Several studies have shown that mpMRI has a high negative predictive value (NPV) for clinically significant disease, the NPV having been estimated at 90% or higher^(14,20,21)^. In addition, mpMRI compares favorably with existing clinicopathologic scoring systems. It has been shown to have higher sensitivity, positive predictive value, and overall accuracy than do several established scoring systems. The overall accuracy of mpMRI for identifying clinically significant disease is reported to be 92%^(21)^, compared with only 70% for the D’Amico criteria, 88% for the Epstein criteria, and 59% for the Cancer of the Prostate Risk Assessment system.

In patients eligible for AS according to Prostate Cancer Research International Active Surveillance criteria^(6)^, a visible lesion on MRI strongly predicts significant PCa. Similarly, a PI-RADS 5 lesion has been shown to be associated with upstaging and an unfavorable outcome^(22)^. In addition, the apparent diffusion coefficient (ADC) values on DWI and the PI-RADS v2 score have been shown to independently predict which patients have or do not have clinically significant disease^(23)^.

Although all of these results support the use of MRI as a PCa biomarker and a criterion for AS eligibility, at least two retrospective studies reported that MRI has a low NPV, which would limit its value in the management of cases in men under AS^(22,24)^. However, one of those studies used only T2-weighted images^(24)^, a protocol that is not representative of the current standards for mpMRI^(25)^.

### Follow-up of patients under AS

Every AS protocol involves periodic risk re-assessment to identify progression of disease and possible upgrading, which is seen in up to 35% of men with clinically localized PCa in extended follow-up. Monitoring of patients in AS includes routine serum PSA measurements two to four times a year and prostate biopsy every one or two years^(21)^. Although PSA and PSA kinetics are frequently used to monitor patients, neither is considered reliable enough to determine changes in therapeutic planning toward any intervention, surgery, or radiation/hormonal therapy. Prostate biopsy is still most often performed with transrectal ultrasound (TRUS) guidance, which has well-established limitations.

The incorporation of serial MRI into AS protocols has been recently advocated to address the limitations of PSA and TRUS-guided biopsy, because MRI can identify new lesions or progression of visible lesions, such as changes in tumor size or vascularity and the development of extraprostatic extension^(18,26)^. Several studies have suggested that MRI could be used to determine the need for and timing of biopsy^(14,18,20,27-30)^. Nevertheless, this remains a controversial topic and the major international guidelines still recommend biopsy at regular intervals. However, in patients with low-risk disease and stable findings on mpMRI and biopsy, it seems reasonable to replace routine biopsy with MRI follow-up^(31)^. Stable lesions on mpMRI are associated with Gleason score stability, as demonstrated by Walton Diaz et al.^(28)^, and a persistently negative MRI result suggests stable low-grade disease^(32,33)^. The reported specificity and NPV of MRI for PCa upgrading is consistently high across studies^(28,30,31,34,35)^. That implies that a negative MRI result provides strong evidence of a lack of upgrading or reclassification of disease, potentially allowing the interval between surveillance biopsies to be increased.

**Figure 1:**
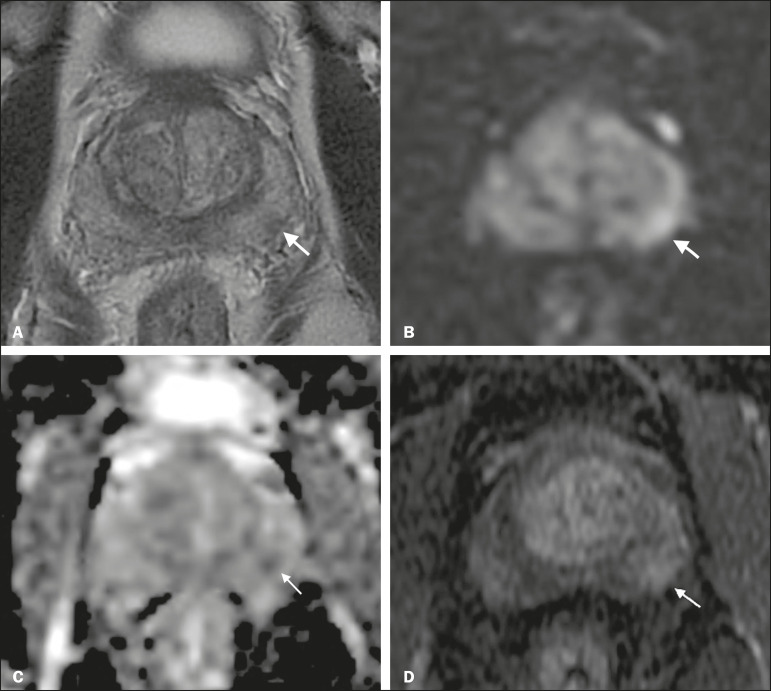
A 62-year-old patient with PCa classified as International Society of Urological Pathology (ISUP) grade 1 (Gleason score of 6) in 2 of 12 cores in a systematic biopsy. After he had been in an AS protocol for one year, mpMRI was requested. **A:** Axial T2-weighted image showed a small, ill-defined hypointense area. **B,C:** DWI (b = 1400 mm/s^2^) and ADC map showing a small focus of restriction at the same location identified on the T2-weighted image. **D:** Dynamic contrast-enhanced imaging showing early enhancement of the same area. The patient was withdrawn from AS, operated on and an ISUP 3 (Gleason score of 4+3) was found at surgery.

**Figure 2:**
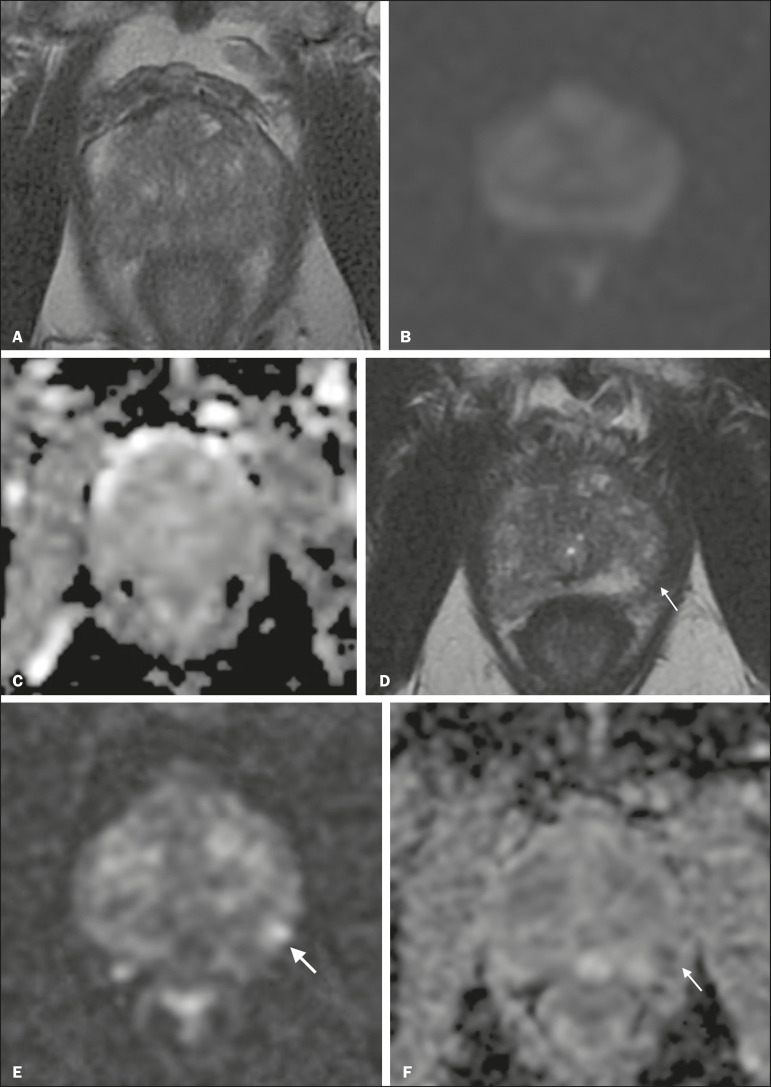
A 54-year-old patient with PCa, classified as International Society of Urological Pathology (ISUP) grade 1 (Gleason score of 6) in 2 of 12 cores in a systematic biopsy, who underwent MRI prior to being enrolled in an AS protocol (in January of 2018). Axial T2-weighted image (**A**), DWI (**B**), and ADC map (**C**), showing no definite lesions. One year later (in January of 2020), another MRI was requested. **D:** Axial T2-weighted image showing a small but well-defined hypointense area in the left peripheral zone. **E:** DWI showing a bright area in the same location. **F:** ADC map showing a small area of restricted diffusion in the same location. A clinically significant lesion was suggested and later confirmed.

Patients with a visible tumor on MRI tend to be less well suited for AS than are those without suspicious lesions on MRI^(33,34)^. Various studies have shown that a suspicious lesion on MRI carries a significant risk of disease reclassification on biopsy^(14,20,27)^, as detailed in Figures 1 and 2. That is particularly true for new lesions assigned a PI-RADS score of 4 or 5, although also some that are categorized as PI-RADS 3^(36)^.

**Figure 3:**
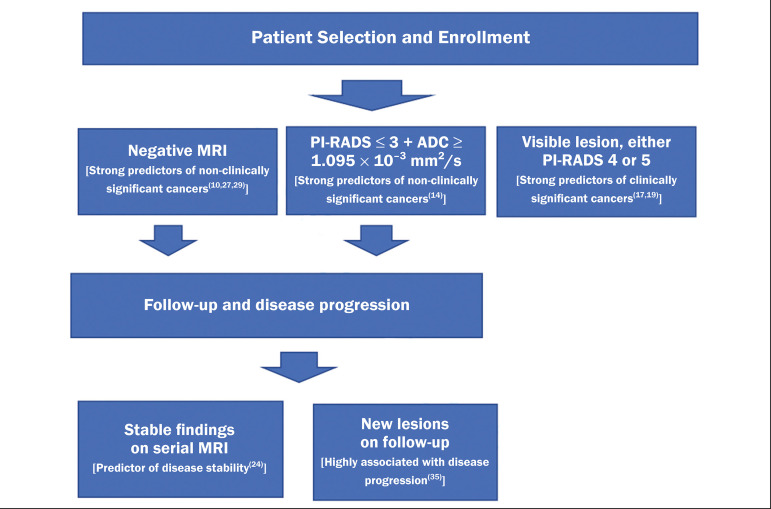
Main findings on MRI examinations and their significance in patients enrolled in AS protocols.

It has been demonstrated that mpMRI can predict reclassification of men considered eligible to AS. Patients with a greater number of visible lesions and higher PI-RADS scores are more likely to be classified outside AS criteria after confirmatory biopsy^(14,22)^. In addition, MRI is very important in the evaluation of men who present with persistently elevated serum PSA and negative TRUS-guided biopsy. Furthermore, mpMRI localizes suspicious lesions in the gland, which can then be targeted under MRI guidance using in-bore, fusion, or cognitive biopsy approaches. In particular, MRI is good at identifying anterior tumors and apical tumors that are often not sampled during a TRUS-guided procedure^(37)^. The cancer detection rate is higher for MRI-targeted biopsy than for TRUS-guided biopsy^(35)^. Because MRI-targeted biopsy requires fewer sample cores^(28,32,38,39)^, it should be utilized more often in patients under AS to overcome the limitations of the traditional random biopsy techniques. By reducing the frequency of repeat biopsy, MRI may be a cost-effective option for patients under AS. A cost-effectiveness modeling study revealed that the number of discounted quality-adjusted life-years was higher for MRI/ultrasound fusion biopsy than for mpMRI alone, mpMRI with biopsy, and TRUS-guided biopsy^(40)^. The findings on MRI examinations and their significance in patients in AS protocols are summarized in Figure 3. Nevertheless, a few studies have indicated that the addition of MRI with targeted biopsies to systematic biopsies did not significantly increase upgrading in comparison with systematic biopsy alone in men under AS^(39)^. Despite several studies showing the ability of MRI to identify disease progression in men under AS, there are yet no clearly defined criteria for disease progression on MRI.

### Limitations of MRI in AS - the unanswered questions

#### Variations in the NPV of MRI

The NPV described for MRI ranges from 80% to 100%^(14,20,21)^, which suggests that MRI does not completely exclude high-grade cancer. In some cases, that might be because the tumor was invisible, whereas in other cases it could be because the radiologist did not see the tumor or because it was masked by or mimicked a benign tumor. In such cases, if the MRI technique and interpretation improve, MRI may be more easily incorporated into AS^(41)^.

#### Lack of consensus among the existing international guidelines on the use of MRI in AS protocols for the enrollment and follow-up of patients

Some of the existing international guidelines for AS (Table 2), including those of the National Comprehensive Cancer Network, British Association of Urological Surgeons, and American Urologist Association, support the use of mpMRI, together with PSA measurement, digital rectal examination, and TRUS-guided biopsy, to determine the suitability of patients for AS and the appropriate follow-up of those patients^(42)^. In contrast, some other guidelines for AS, such as the Prostate Cancer Research International: Active Surveillance criteria, as well as the guidelines of the European Association of Urologists, the Canadian Urological Association, Cancer Care Ontario, and the National Institute for Health and Care Excellence, have not included mpMRI as part of the criteria for the selection of patients for AS and their appropriate follow-up^(42)^. This lack of consensus may be due to the level of skill required for the accurate interpretation of mpMRI scans, which only some radiologists now possess. The lack of such skill may result in a low level of confidence in a negative MRI result^(43)^.

**Table 2:** International guidelines for AS.

Guideline	PSA (ng/mL)	PSA density (ng/mL^2^)	Positive biopsy cores (n or %)	Gleason score	Clinical T stage	mpMRI
NCCN	< 10	-	-	*<* 6	T1-T2a	Yes
BAUS	< 10	-	< 50%	*< 6*	T1-T2	Yes
AUA	< 10	-	-	*< 6*	T1-T2a	Yes
PRIAS	< 10	< 0.2	1-2	*< 6*	T1c/T2	No
EAU	< 10	-	-	*< 6*	T1-T2a	No
CUA	< 10	-	-	*< 6*	< T2a	No
CCO	-	-	-	-	-	No
NICE	< 10	-	-	*< 6*	T1-T2a	No

NCCN, National Comprehensive Cancer Network; BAUS, British Association of Urological Surgeons; AUA, American Urological Association; PRIAS, Prostate Cancer Research International: Active Surveillance; EAU, European Association of Urologists; CUA, Canadian Urological Association; CCO, Cancer Care Ontario; NICE, Na­tional Institute for Health and Care Excellence.

#### Systematic biopsy sampling when lesions are identified on MRI

The use of MRI has improved the diagnosis of PCa and MRI targeted biopsy has been shown to have an advantage over systemic biopsy in patients with clinical suspicion of PCa that have not undergone prior biopsy^(44)^. Following the identification of prostatic lesions on MRI, there are three approaches to target MRI biopsy, namely cognitive fusion biopsy, TRUS-MRI fusion biopsy, and MRI-guided in-bore biopsy. Cognitive fusion biopsy is the least accurate, operator dependent and lack standardization. In contrast, MRI-guided in-bore biopsy, performed under MRI guidance with direct visualization of the lesion, is the most accurate method for the detection of clinically significant cancer. It is, however, costly, and the procedure takes a long time. The third approach, TRUS-MRI fusion biopsy involves the registration and fusion of previously acquired MRI sequences with real-time TRUS images. It is faster than is MRI-guided in-bore biopsy and can be easily performed alongside systematic biopsy. However, TRUS-MRI fusion biopsy has certain limitations, including registration errors and high initial costs^(18,45)^.

Some authors have suggested that targeted biopsy alone should be used, whereas others have stated that targeted biopsy is preferred to systematic biopsy in cases in which a lesion is identified on MRI^(35,46)^, as well as that follow-up with MRI will identify progression and maintain the window of opportunity for cure open, because the tumors will be small^(18,26,31)^. However, when a lesion is identified on mpMRI, some patients may have disease in other areas of the prostate and the Gleason score or grade may be higher. It has also been said that targeting biopsies to abnormal regions of the prostate, as identified on mpMRI, detects a high proportion of clinically significant PCas and may result in lower rates of diagnosis of clinically insignificant tumors^(18,26)^. Systemic biopsy has been recommended in patients with high clinical suspicion PCa and a negative MRI result^(47)^. When systemic biopsy reveals a high Gleason score or grade, treatment should follow the appropriate course.

#### Definition of disease progression on MRI

There are no precise criteria for disease progression on MRI in patients under AS. However, the appearance of a new lesion in patients under AS is a strong predictor of disease progression^(18,26)^, as illustrated in Figure 2. There are other imaging features that could also be helpful, such as increased tumor size and a higher degree of restricted diffusion of DWI, although even those parameters have their limitations. Subtle changes in size may reflect real variations or inter- or intra-reader variation. To an even greater effect, changes in ADC values can be due to real changes in lesion structure or to reader variation, as well as to the use of different scanners^(48)^.

There are conflicting reports on the sensitivity and positive predictive value of mpMRI in the prediction of disease upgrading and tumor progression^(20,28,34,35)^. As previously stated, the appearance of a new lesion in patients during follow-up for AS is a strong predictor of disease progression. However, there is yet no consensus regarding the diagnostic accuracy of serial mpMRI for the detection of clinically significant neoplasia and more data are required to elucidate this issue. For instance, for patients in an AS protocol with visible lesions on mpMRI, could a decrease in ADC (assuming a stable lesion size) be considered disease progression? If so, what would be the threshold value for that? Some studies have also shown that PCa detection with mpMRI alone is not yet sensitive enough to omit systematic biopsy during follow-up after an initial 12-core TRUS-biopsy^(22)^.

## CONCLUSION

In this review article, we have summarized the strengths and limitations of MRI for determining the eligibility of patients for AS and defining the appropriate follow-up. In the selection of patients for AS enrollment, mpMRI has a high NPV and high specificity, which can likely reduce the misclassification rate of clinically significant PCas. During AS follow-up, the time from referral to MRI/ultrasound biopsies, MRI-suspicion score, and MRI total lesion density among other factors are significantly associated with tumor upgrading, as are other factors. In men under AS for low-risk PCa, suspicious lesions on mpMRI are associated with a substantial increase in subsequent upgrading.
